# Rescue and evaluation of a recombinant PRRSV expressing porcine Interleukin-4

**DOI:** 10.1186/s12985-015-0380-7

**Published:** 2015-11-14

**Authors:** Zhijun Li, Gang Wang, Yan Wang, Chong Zhang, Xinglong Wang, Baicheng Huang, Qiongyi Li, Liangliang Li, Biyun Xue, Peiyang Ding, Shahid Faraz Syed, Chengbao Wang, Xuehui Cai, En-Min Zhou

**Affiliations:** Department of Preventive Veterinary Medicine, College of Veterinary Medicine, Northwest A&F University, Yangling, Shaanxi 712100 China; Scientific Observing and Experimental Station of Veterinary Pharmacology and Veterinary Biotechnology, Ministry of Agriculture, Yangling, Shaanxi 712100 China; State Key Laboratory of Veterinary Biotechnology, Harbin Veterinary Research Institute of Chinese Academy of Agriculture Science, Harbin, 150001 China

**Keywords:** PRRSV, Reverse genetic technology, Porcine IL-4, Vaccine

## Abstract

**Background:**

The current vaccines for porcine reproductive and respiratory syndrome virus (PRRSV) have failed to provide broad protection against infection by various strains of PRRSV. Porcine Interleukin-4 (pIL-4) plays an important role in the regulation of the immune response and has been used previously as an immunological adjuvant. The objective of this study was to construct a recombinant PRRSV expressing pIL-4 and to evaluate the immune response of the recombinant virus in piglets.

**Methods:**

The pIL-4 gene was inserted in the PRRSV (CH-1R strain) infectious clone by overlap PCR. Indirect immunofluorescence assay (IFA) and Western blotting were used to confirm the recombinant virus. The stability of the recombinant virus was assessed by DNA sequencing and IFA after 15 passages *in vitro*. Recombinant virus was injected into pigs and efficacy of immune protection was evaluated in comparison with the parental virus.

**Results:**

The recombinant virus (CH-1R/pIL-4) was successfully rescued and shown to have similar growth kinetics as the parental virus. The recombinant virus was stable for 15 passages in cell culture. Pigs vaccinated with CH-1R/pIL-4 produced a similar humoral response to the response elicited by parental virus, but IL-4 level in the supernatant of PBMCs from pigs vaccinated with CH-1R/pIL-4 was significantly higher than the parent virus at 28 days post-immunization (DPI). Flow cytometric (FCM) analysis showed that the percentage of CD4^+^CD8^+^ double positive T (DPT) cells in the CH-1R/pIL-4 vaccinated group was significantly higher than the parental virus at 3 and 7 Days Post-Challenge (DPC), and the IL-4 level in the blood significantly increased at 7 DPC. However, the viral load and histopathology did not show significant difference between the two groups.

**Conclusions:**

A recombinant PRRSV expressing porcine IL-4 was rescued and it remained genetically stable *in vitro*. The recombinant virus induced higher DPT ratios and IL-4 levels in the blood after HP-PRRSV challenge compared to the parental virus in piglets. However, it did not significantly improve protection efficacy of PRRSV vaccine.

## Background

Porcine reproductive and respiratory syndrome (PRRS) is characterized by clinical signs of reproductive failure in pregnant sows and respiratory distress in pigs of all ages [1]. Since it’s first reported in 1987, PRRS has become a severe threat to the swine industry worldwide [2]. The causative agent of PRRS, the porcine reproductive and respiratory syndrome virus (PRRSV) was successfully isolated in EU and USA in 1991 and 1992, respectively [3, 4]. Surprisingly, the two genotypes showed sequence identity of only 60 % and a high degree of antigenic variation [5, 6]. In June 2006, an unparalleled, large-scale, PRRS outbreak occurred in China, leading to widespread infection which continues to be a serious problem today. Several studies have confirmed that a unique highly pathogenic strain of PRRSV (HP-PRRSV) was responsible for this outbreak [7–9].

PRRSV is an enveloped virus with a single-stranded, positive-sense RNA genome of approximately 15 kb in length [10]. The genome contains a cap structure at the 5′ end, a poly (A) tail at the 3′ end, and 5′ and 3′ un-translated regions (UTRs). Two overlapping open reading frames (ORF 1a and ORF 1b) occupy two thirds of the genome at the 5′ end. These ORFs encode two large polyproteins that undergoes co-translational and post-translational processing to give rise to at least 14 non-structural proteins (nsps). ORFs 2–7 code for viral structural proteins GP2, E, GP3, GP4, GP5a, GP5, M, and N, respectively [11–14]. According to the recently accepted replication model for viruses of the order *Nidovirales* , PRRSV and other arteriviruses synthesize a nested set of subgenomic (sg) mRNAs as a mechanism to regulate the expression of structural or accessory proteins [15]. Base pair interactions between the transcription-regulating sequences (TRSs) located at the 3′ end of the leader sequence and the complement of body TRSs presented upstream of each gene, have been shown to be essential for this process [16].

It is well recognized that due to the genetic diversity of the PRRSV strains, none of the current vaccines can completely protect against PRRSV infections. For PRRSV vaccines, the efficacy of protective immunity is usually assessed by the reduction in viremia after challenge with a virulent virus [17, 18]. Sufficient amount of neutralizing antibodies may prevent the infection, but cannot clear the virus in blood during the course of the infection. Therefore, vaccine immunity relies on the induction of the protective cell-medicated immune responses in prevention against PRRSV infection [19, 20]. CD4^+^CD8^+^ double positive T (DPT) cells are the main effectors of adaptive cellular immune response and essential to clear viral infection [21]. IFN-γ secreting cells are mainly DPT cells and whose levels are correlated with the host ability prevent PRRSV infection [22].

It is clear that there is an urgent need for a safe and more effective PRRSV vaccine. In order to increase the efficacy of the vaccine, an alternative approach is to co-deliver cytokines to up-regulate the immune response of vaccine antigens. Interleukin-4 (IL-4) is a pleiotropic cytokine and has been used as an adjuvant to enhance immune response of PRRSV vaccines [23, 24]. In addition, IL-4 may positively modulate vaccination mediated clearance of PRRSV [22, 25]. In this study, porcine Interleukin-4 (pIL-4) gene was inserted between the N gene and the 3′-UTR of a live attenuated PRRSV infectious clone, along with a copy of the transcription regulating sequence for ORF6 (TRS6), to create a recombinant PRRSV. The growth characterization and genetic stability of the recombinant virus was first analyzed *in vitro*. Further, recombinant virus was injected into piglets to evaluate its potential role for improving vaccine efficacy in protection against HP-PRRSV infection.

## Results

### Rescue of recombinant virus harboring pIL-4

**Fig. 1:**
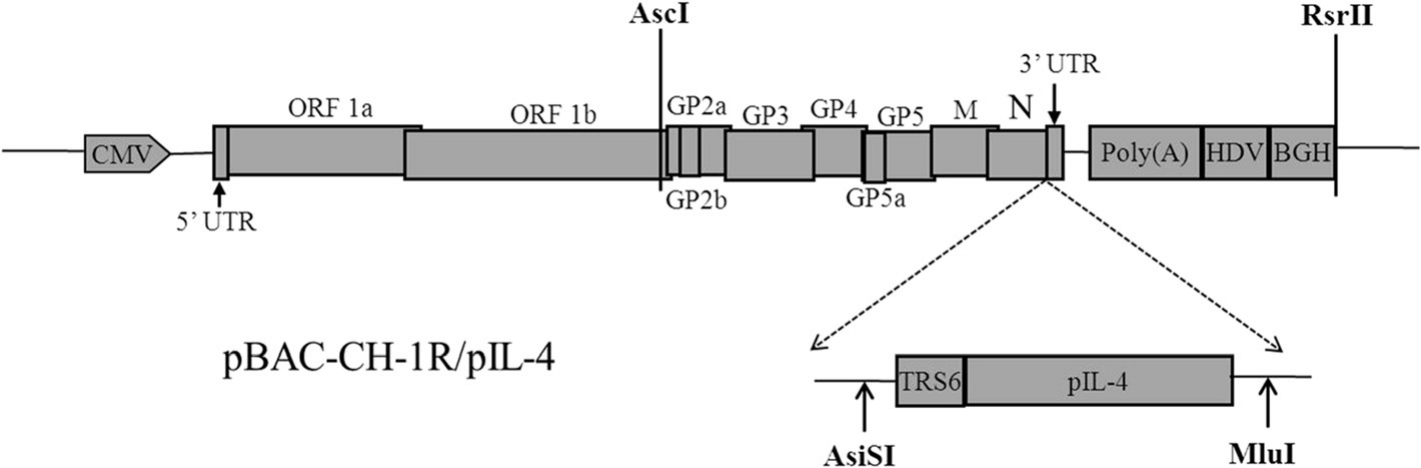
Schematic diagram of the recombinant plasmid (pBAC-CH-1R/pIL-4). The porcine IL-4 (pIL-4) gene was inserted between the N gene and the 3′-UTR of the attenuated PRRSV infectious clone (pBAC-CH-1R). It exhibited expression as a separate sub-genomic RNA with a copy of the transcription regulation sequence for ORF6 (TRS6)

The recombinant plasmid pBAC-CH-1R/pIL-4 (Fig. 1) was constructed according to the primers as shown in Table 1. The recombinant virus CH-1R/pIL-4 was successfully rescued after transfecting pBAC-CH-1R/pIL-4 plasmid into Marc-145 cells. CPE were markedly observed after 3 days post-transfection (data not shown), supernatants were then harvested and passaged 3 times in Marc-145 cells.

**Table 1:** Primers used for construction and identification of recombinant PRRSV infectious clones

Primers	Nucleotide sequence (5'-3')	Sense
F1-1	TTAACCAGAGTTTCAGCGGAACAATGGGTCTCACCTCCCAAC	+
F1-2	*GCGATCGC*TGATGGTTCCGTGGCAACCCCTTTAACCAGAGTTTCATG	+
R1	CG*ACGCGT*CGTCAACACTTTGAGTATTTCTCCTT	-
F2	ATACTGTGCGCCTGATCCGC	+
R2	TCGCCAATTAAACTTTACCCCCACA	-

The italics in the sequence denote the restriction enzyme sites (*Asis* I, *Mlu* I)

**Fig. 2:**
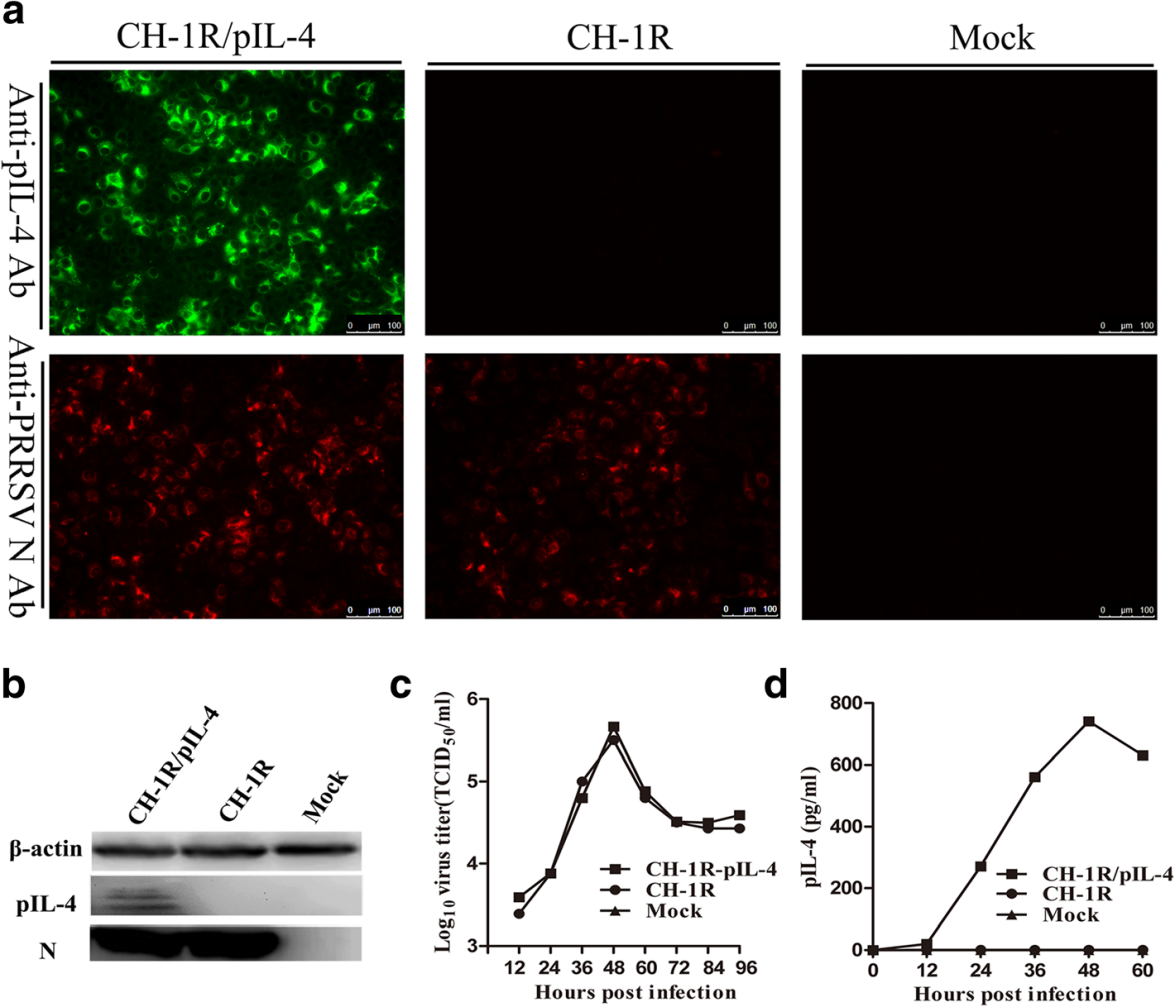
Characterization of recombinant PRRSV expression of the pIL-4 gene in Marc-145 cells. **a** IFA detection of pIL-4 and N expression in Marc-145 cells infected with parental virus or recombinant virus. Original magnification 200×. **b** Western blot detection of pIL-4 and N protein in cell lysates of Marc-145 cells infected with recombinant virus and parental virus. **c** Growth curves of the recombinant and the parental virus in Marc-145 cells. **d** Kinetics of pIL-4 accumulation in the supernatants of Marc-145 cells infected with the recombinant virus vs. parental virus or mock control. Data are represented as the mean of three independent experiments

The presence of recombinant PRRSV was identified by indirect immunofluorescence assay (IFA) and western blotting using the PRRSV N-specific antibody (6D10). Expression of pIL-4 was confirmed using a goat anti-pIL-4 polyclonal Ab. As shown in Fig. 2a, the presence of the virus was confirmed by the visualization of red fluorescence, and pIL-4 with green fluorescence. Western blotting was used to verify protein expression of N and IL-4 (Fig. 2b).

As expected, pIL-4 expression via an additional TRS did not influence the growth characteristics of the virus. As shown in Fig. 2c, growth rate and maximum titer of the recombinant virus were similar to those of the parental virus. Titers peaked at 48 h post infection (hpi) for both viruses.

The expression of pIL-4 was further analyzed by determining the accumulation kinetics of pIL-4 in the supernatant of infected Marc-145 cells. The concentration of IL-4 was 300 pg/mL at 24 hpi, and it reached the maximum concentration of 800 pg/mL at 48 hpi (Fig. 2d).

### Genetic stability testing

The recombinant virus was serially passaged in Marc-145 cells for 15 times to examine the genetic stability of pIL-4 in the virus over time. Viral RNA was isolated from cells after passage 5, 10 and 15 post-infection with recombinant virus. The presence of the pIL-4 gene in the viral genome was confirmed using RT-PCR. Unexpected gene insertion, deletion and mutation were ruled out by sequencing (data not shown).

### Humoral immune responses

**Fig. 3:**
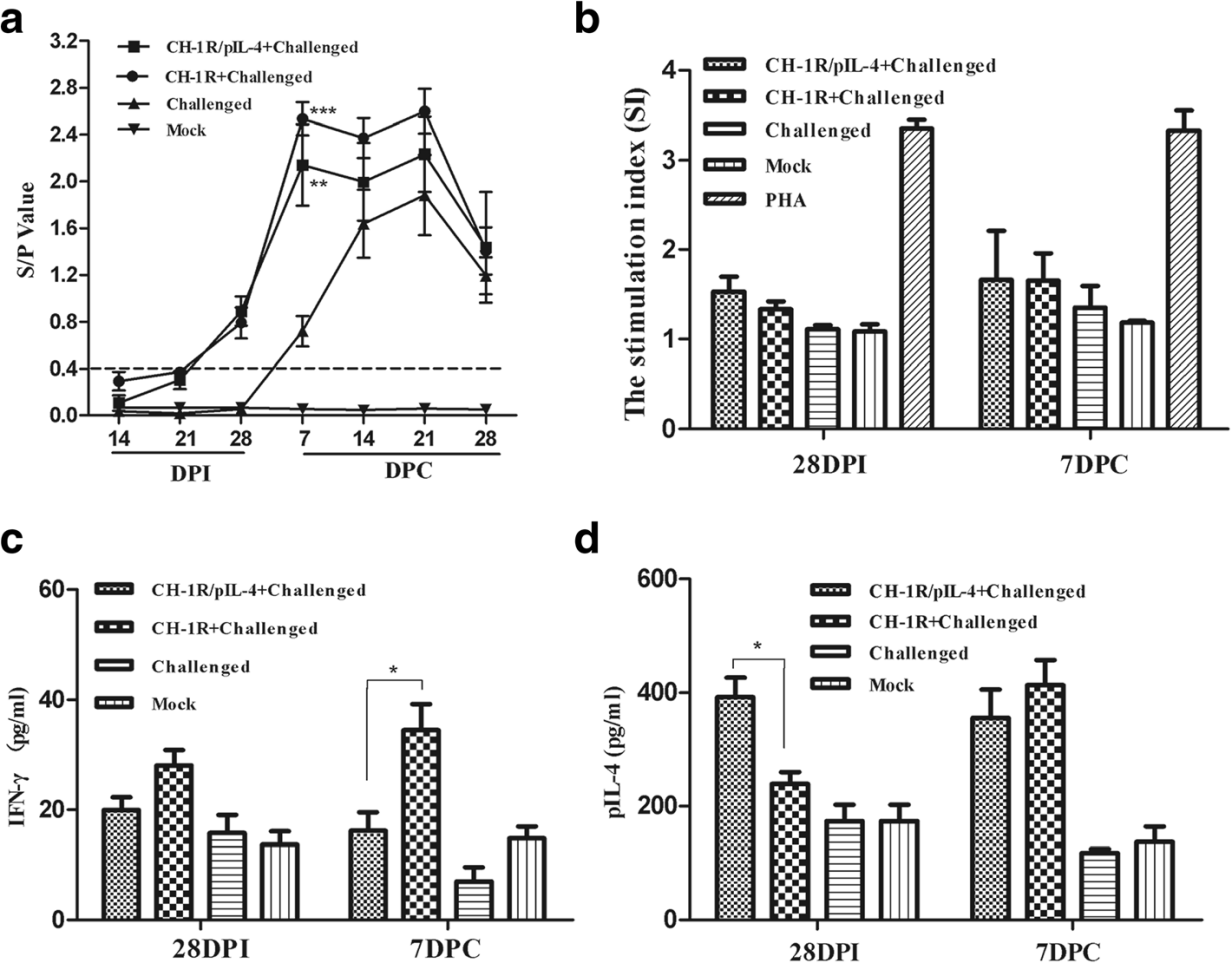
Humoral and cell-mediated immunity in pigs infected with the recombinant and the parental virus. **a** PRRSV-specific serum antibodies were measured with the IDEXX HerdChek® PRRSV ELISA 2XR Kit. S/P ratios of greater than 0.4 are considered positive. S/P = (Sample A (650)-Negative Control) / (Positive Control- Negative Control). Parental and recombinant virus groups were compared to the challenged control group. **denotes *p* < 0.01; *** represents *p* < 0.001. **b** PBMCs were collected from the piglets stimulated with purified PRRS virions. PHA was the positive control. After 72 h of stimulation, the cells were treated with WST-8 for 2 h and the OD_450_ values were determined to calculate the stimulation index (SI). IFN-γ(**c**) and IL-4 (**d**) concentrations in the supernatants of PBMC cultures were tested after 72 h of stimulation using commercial ELISA Kits. *indicates p < 0.05. Data represent the mean ± SD of five pigs from each group

The host humoral immune response was assessed in pigs at various time-points (Fig. 3a). The antibody titers of the pigs in the two vaccinated groups showed similar trend and they could be initially detected at 21 DPI. Titers significantly increased at 7 through 21 DPC and then dropped at 28 DPC. Antibodies for the challenged (non-vaccinated) control group were initially detected at 7 DPC. The titers were markedly increased by 21 DPC and then dropped at 28 DPC. Virus titers were significantly higher at 7 DPC in challenged unvaccinated group than in the groups vaccinated with parental or recombinant virus (*p* < 0.01, *p* < 0.001, respectively). No seroconversion was evident in the mock group.

### T lymphocyte proliferation assay

Purified PRRSV CH-1R antigen was used as stimulator to perform lymphocyte proliferation assay. As shown in Fig. 3b, the CH-1R/pIL-4 vaccinated group stimulated PRRSV-specific lymphocyte proliferative response which was similar to the response of the CH-1R vaccinated group at 28 DPI and 7 DPC.

We further monitored the temporal expression of INF-γ and IL-4 in PBMCs after stimulation with purified PRRSV. As shown in Fig. 3c, IFN-γ expression in PBMCs from pigs in the CH-1R/pIL-4 group was significantly lower than expression in the CH-1R vaccinated group at 7 DPC (*p <* 0.05). However, IL-4 expression was significantly higher in the CH-1R/pIL-4 group than in the CH-1R group at 28 DPI (*p <* 0.05) (Fig. 3d).

### T lymphocyte phenotypic analysis

The percentages of CD3^+^ single positive T (SPT) cells, CD4^+^ SPT cells, CD8^+^ SPT cells, and CD4^+^ CD8^+^ double positive T (DPT) cells in peripheral blood were measured by flow cytometric (FCM) analysis. The percentage of CD3+ SPT cell subpopulations remained relatively stable in all groups during the experimental period. There was no significant change in the levels of the DPT cells in either of the immunized groups during vaccination period, however, the numbers of DPT cells in the recombinant virus group were markedly higher at 3 and 7 DPC compared with the parental virus group (*p <* 0.05). Interestingly, the DPT cells in the challenged control group rapidly decreased after challenge, returned to normal by 21 DPC. Table 2 shows the complete results of the FCM analysis.

**Table 2:** Flow cytometric analysis for proportion of T lymphocyte subpopulations in peripheral blood of pigs

Marker	14^a^	28^a^	3^b^	7^b^	14^b^	21^b^	28^b^
**CD3** ^**+**^							
Group1	56.4 ± 4.5	61.1 ± 8.6	65.6 ± 3.6	66.4 ± 5.0	57.7 ± 4.7	58.7 ± 7.0	69.8 ± 7.9
Group2	51.9 ± 5.6	49.0 ± 6.7	53.9 ± 6.8	59.9 ± 3.9	51.8 ± 8.4	54.4 ± 5.8	58 ± 6.6
Group3	52.7 ± 3.9	49.8 ± 10.9	44.76 ± 6.3	59.6 ± 8.8	61.1 ± 5.2	57.2 ± 7.3	51.4 ± 8.3
Group4	50.8 ± 2.1	49.8 ± 6.4	48.3 ± 7.0	52.6 ± 8.7	51.8 ± 10.7	50.6 ± 9.7	52.4 ± 6.8
**CD4** ^**+**^							
Group1	18.6 ± 3.4	15 ± 4.1	9.7 ± 3.8	4.6 ± 0.3	4.3 ± 4.2	1.9 ± 0.6	4.1 ± 1.2
Group2	17 ± 1.2	16.5 ± 8.4	5.9 ± 2.9	5.2 ± 5.4	6.3 ± 2.3	1.33 ± 0.9	4.2 ± 2.5
Group3	28.7 ± 0.2	24.9 ± 8.2	11.6 ± 5.5	3.1 ± 1.5	8.0 ± 5.3	20 ± 6.3	22.7 ± 2.2
Group4	23.6 ± 4.3	30.7 ± 5.4	26.5 ± 3.7	32.4 ± 0.2	26.3 ± 3.5	19.6 ± 2.8	23.1 ± 3.3
**CD8** ^**+**^							
Group1	30.3 ± 5.3	33.3 ± 8.1	27.6 ± 6.5	64.8 ± 8.2	50.7 ± 6.6	53.0 ± 9.2	46 ± 0.2
Group2	27.5 ± 5.4	35.5 ± 7.3	26.4 ± 3.0	58.8 ± 9.1	48.6 ± 2.0	51.8 ± 11.2	50.1 ± 4.7
Group3	28.0 ± 4.5	29.1 ± 7.5	13.5 ± 2.1	24.5 ± 6.2	29.7 ± 9.5	38.8 ± 3.2	30 ± 3.6
Group4	29.7 ± 6.4	32.7 ± 3.0	29.3 ± 2.6	29.5 ± 8.7	30.2 ± 8.9	32.4 ± 3.3	36.7 ± 0.5
**CD4** ^**+**^ **CD8** ^**+**^							
Group1	29.4 ± 7.8	23.9 ± 8.6	32.9 ± 3.1*	39.1 ± 2.6*	28.3 ± 7.1	25.4 ± 6.5	31.4 ± 7.7
Group2	30.5 ± 4.2	21.7 ± 9.7	18.3 ± 5.2	26.4 ± 4.5	17.2 ± 7.4	26.0 ± 7.9	29.5 ± 3.8
Group3	18.9 ± 0.1	18.5 ± 7.0	8.7 ± 3.0	7 ± 5.7	13.2 ± 1.2	19.2 ± 6.9	18.1 ± 5.3
Group4	20.5 ± 4.3	22.8 ± 3.1	28 ± 4.8	26.4 ± 10.2	25.7 ± 8.0	23.5 ± 6.5	28.9 ± 4.1

Results are expressed as mean (%) ± SD. Group 1: CH-1R/pIL-4 + Challenged; Group 2: CH-1R + Challenged; Group 3: Challenged; Group 4: Mock. *p < 0.05

^a^Days post- immunization (DPI); ^b^Days post-challenge (DPC)

### Cytokine responses

**Fig. 4:**
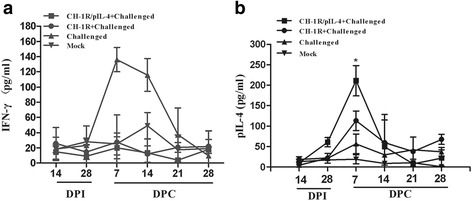
Concentrations of Th1-type (IFN-γ) and Th2-type (IL-4) cytokines in the sera of pigs. Serum samples were collected from five pigs in each group at different time points and quantitative ELISA kits were used to detect level of IFN-γ (**a**) or IL-4 (**b**). The CH-1R vaccinated group was compared to the recombinant vaccinated group and * denotes *p <* 0.05. Data represent the mean ± SD of five pigs from each group

At 14 DPI and 0, 7, 14, 21 and 28 DPC, the sera of the pigs were collected to detect the production of IFN-γ and IL-4. As shown in Fig. 4a, IFN-γ expression in sera was not significantly different between two immunized groups during experimental period. However, mean IL-4 level in the serum of group vaccinated with recombinant virus was significantly higher than that of parental virus group at 7 DPC (*p <* 0.01) (Fig. 4b).

### Observation of clinical signs and rectal temperature testing

**Fig. 5:**
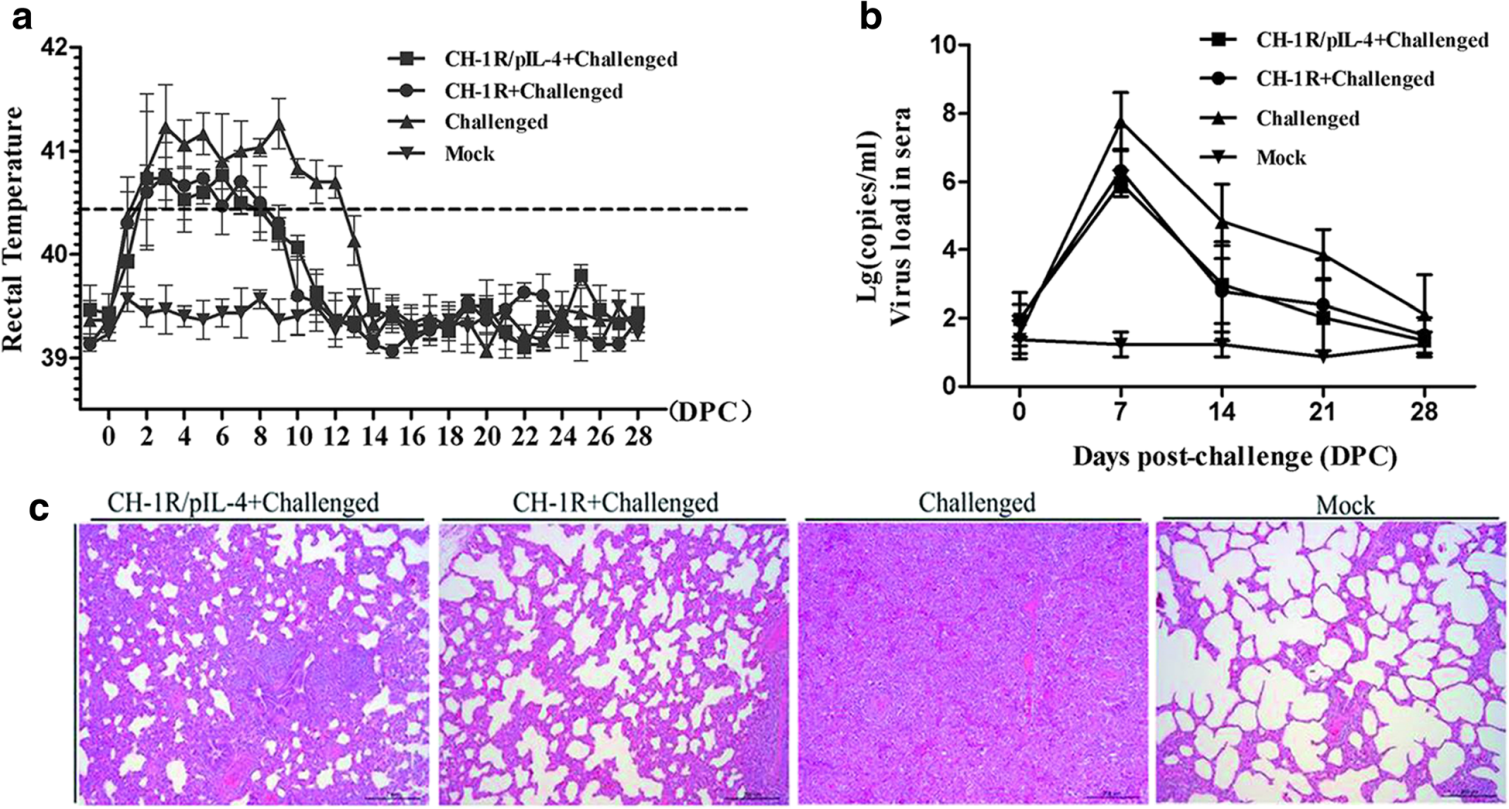
Rectal temperatures, viremia assessment and pathological examination of five pigs in each group challenged with HP-PRRSV (HuN4). **a** Rectal temperatures were recorded after challenge; **b** Viral RNA in serum was detected at 0, 7, 10, 14, 21 and 28 DPC; **c** The pigs were sacrificed at 28 DPC, and lung tissue samples were examined using hematoxylin and eosin staining (H&E). Data are shown as mean ± SD for five pigs per group; Scale bar: 200 μm

All of the pigs presented with a high fever (≥40 °C) after challenge. Rectal temperatures from the challenged group increased rapidly at 2 DPC and remained above 41 °C from 3 to 12 DPC, before returning to normal at 14 DPC. Two immunized groups also showed similar fever pattern, but the overall rectal temperatures were relatively low and of shorter duration. There was no significant difference in fever magnitude and duration between groups. No rectal temperature variations were observed in the mock group during the experimental period (Fig. 5a).

The challenged group showed obvious clinical signs of infection, including dyspnea, severe lethargy, cough, and anorexia after challenge. Two pigs showed typical “blue ears” and died at 7 and 10 DPC. Similar clinical signs were observed in the two immunized groups following challenge, but the signs were milder and of shorter duration relative to the unimmunized pigs. The scores of clinical signs between two immunized groups were not significant difference (*p* > 0.05), but were significantly lower than that in the challenged control group (*p* < 0.05) (Table 3).

**Table 3:** The scores of clinical signs after challenge and lung lesions recorded at 28 DPC^a^

Groups	Scores of clinical signs (±SD)^b^	Scores of lung lesions (±SD)^c^
CH-1R/pIL-4 + Challenged	3.9 ± 0.18B	21.5 ± 3.32B
CH-1R + Challenged	3.4 ± 0.27B	23.6 ± 5.57B
Challenged	5.6 ± 1.58A	51.2 ± 8.33A
Mock	3 ± 0.00B	6.67 ± 0.58C

^a^Within each column, the different letters (A, B, C) represent the values significantly different (*p* < 0.05)

^b^Scores of clinical sign were expressed as the sum of daily observations of all clinical signs

^c^Percentage of the entire lung affected by pneumonia

### Viremia assessment

At 0, 7, 14, 21 and 28 DPC, the blood samples of the pigs were collected and the viral loads in the sera were investigated. As shown in Fig. 5b, all of the challenged groups developed highest degree of viremia at 7 DPC, gradually dropped at 14 DPC and then returned to normal. The two immunized groups exhibited slightly less viremia than the un-immunized challenge group, but no significant difference was observed (*p >* 0.05).

### Pathological examination

On histological examination, lung lesions of pigs in the challenged group exhibited alveolar epithelial cell proliferation and extensive infiltration of inflammatory cells. However, the interstitial pneumonitis observed in the CH-1R/pIL-4 or CH-1R vaccinated groups were milder than that of un-immunized challenged group at 28 DPC. The mock group was normal (Fig. 5c).

## Discussion

The non-essential regions of the PRRSV nsp2 gene have been utilized previously as an insertion site for expressing a foreign gene [26–31], but stability of the recombinant virus was a concern [32]. As a potential remedy to this problem, foreign gene was inserted between PRRSV ORF1b and ORF2a, along with a copy of TRS. This approach had particular advantage of minimizing the effects of expression and post-translational modification of viral proteins [33], and a series of recombinant PRRS viruses have been constructed using this approach [33–37]. In this study, the pIL-4 gene was inserted between N gene and 3′-UTR of a live attenuated PRRSV infectious clone using a similar strategy. Both recombinant virus and parental virus exhibited similar virus titers after growth in Marc-145 cells, and this pattern was critical for recombinant vaccine development [38].

Previous studies demonstrated that pIL-4 markedly enhanced the protective immune response of pigs and improved the efficacy of the MLV in preventing PRRS disease [23], and pIL-4 can enhance immunogenicity of the PRRSV ORF5 gene vaccine [24]. Conversely, however, pigs that received PRRSV ORF7 DNA vaccine plus pIL-4-encoded plasmids did not develop any improved humoral and cellular immune responses [39]. In present study, recombinant virus expressing pIL-4 did not significantly enhance the protective immune response after vaccination. Thus, observations on the adjuvant effect of pIL-4 on PRRSV vaccine were inconsistent, which may be related to the species. Porcine IL-4 cannot be used as immunological adjuvant since it was reported that IL-4 plays a different role in pigs than in mice and humans, and porcine IL-4 blocks antibody production and also suppresses antigen-stimulated proliferation of B cells [40]. Another potential reason may be attributed to insufficient expression levels of IL-4 or inefficiency of IL-4 in potentiating anti-PRRSV immunity *in vivo*. In the absence of adequate amount, IL-4 cannot work efficiently as adjuvant to enhance immunity efficacy or prevent PRRSV infection [41]. Thus, additional studies on IL-4 as an adjuvant are required in different types of PRRSV vaccine as well as in other veterinary vaccines to evaluate its adjuvant property.

In this study, we monitored the percentage of several T lymphocyte subpopulations in the blood at different time. Our data showed that the frequency of total CD3^+^ T subpopulations of pigs from all groups was relatively stable during experimental period (Table 2). Conversely, a rapid down-regulation of CD4^+^CD8^+^ double positive T (DPT) cells was observed in the challenged control group after HP-PRRSV (HuN4) infection, as previously reported [42, 43]. These results suggest one mechanism of the PRRSV-mediated delay in initiation of virus-specific adaptive immunity is by altering frequency of important lymphocytes early post-infection. In addition, we also observed that DPT cells in the recombinant virus group were markedly higher than the parental virus group at 3 and 7 DPC. Meanwhile, mean IL-4 level in the serum of group vaccinated with recombinant virus was significantly higher than that from parental virus group at 7DPC (Fig. 4b). IL-4 is a mediator of DPT cells cross-talk leading towards the development of immunity against an infectious pathogen [44], and IL-4 had a potent positive influence on the generation of DPT cells originally cloned from CD4^+^CD8^−^ T lymphocytes [45, 46]. Our results also indicated that the higher level of IL-4 may be helpful to increase the DPT ratio in the serum.

IL-4 expression has been shown to control macrophage inflammatory activities in the pig [40], and IL-4 may positively modulate vaccination mediated clearance of PRRSV [22, 25]. In this study, there were significantly higher levels of IL-4 detected in recombinant virus vaccinated pigs in supernatants of PBMCs at 28 DPI (Fig. 3d), and IL-4 was significantly increased in the sera of pigs vaccinated with recombinant virus at 7 DPC (Fig. 4b). However, the viremia did not show significantly difference compared with parental virus after challenge (Fig. 5b). It is possible that the role of IL-4 may be strain dependent, or it may not have a direct correlation in protecting pigs from HP-PRRSV infection.

IFN-γ is a key cytokine that is associated with host cell-mediated immunity (CMI) response, which is secreted by natural killer cells and several different T cell subpopulations [25]. IFN-γ reduces PRRSV infection in porcine alveolar macrophages *in vitro* [47]. A live attenuated PRRSV vaccine that induced high IFN-γ secreting cell frequencies protected pigs against viremia [48, 49]. In this study, piglets immunized with CH-1R strain did not produce higher levels of IFN-γ, conversely, higher levels of IFN-γ were found in piglets infected with HP-PRRSV HuN4 strain (Fig. 4a). Nonetheless, CH-1R strain can offer protection against clinical disease after HP-PRRSV HuN4 infection, and this observation is consistent with our previously published work [50, 51]. Therefore, the role of IFN-γ in the process of the immune protection to CH-1R vaccination is not clear and need further investigation.

## Conclusions

This is first report to demonstrate the use of PRRSV as a vector to express porcine IL-4. The pIL-4 gene was stably expressed and did not affect the replication capability of the parental virus. The recombinant virus could enhance the T cells immune response by increasing IL-4 expression level and DPT cells ratio in the blood of piglets after HP-PRRSV infection. However, it did not significantly improve the efficacy of PRRSV vaccine.

## Materials and methods

### Cells and viruses

Marc-145 cells (CCTCC, Wuhan, China) were used for rescue and passaging of PRRSV. The cells were propagated in Dulbecco’s Modified Eagle’s medium (DMEM) with 10 % heat-inactivated FBS (Gibco®, USA) at 37 °C with 5 % CO_2_. For virus infection and titration, DMEM supplemented with 3 % FBS was used. HP-PRRSV strain HuN4 (GenBank: EF635006) was used in this study for challenge.

### Construction of plasmids, transfection and virus passage

A PRRSV infectious clone, pBAC-CH-1R, was constructed as previously described [52]. The porcine Interleukin-4 (pIL-4) gene (GenBank: HQ236500.1), with a TRS motif and Asis I/ and Mlu I restriction enzyme sites at the 5′-and 3′- terminal ends, respectively, was constructed using fusion PCR. The PCR fragments were digested and inserted into the pBAC-CH-1R vector. The resulting construct, pBAC-CH-1R/pIL-4 was sequenced to confirm the presence and correct cloning of the desired pIL-4 gene sequence (Fig. 1). The primers used for these assays are shown in Table 1.

The pBAC-CH-1R/pIL-4 plasmid was transfected into Marc-145 cells as described previously [52]. Cell culture supernatants were harvested at 4–5 days post transfection and used to infect fresh Marc-145 cells for virus passage.

### Growth kinetics and expression of pIL-4 of recombinant virus

Marc-145 cells were infected with the recombinant or the parental virus at a multiplicity of infection (MOI) of 0.01. 200 μL supernatant was collected at 12, 24, 36, 48, 72, 84 and 96 h post infection (hpi) and stored at −70 °C. Viral titration was determined using the Reed-Muench method. The expression of pIL-4 was determined using a commercial porcine IL-4 ELISA kit (Cloud-Clone Corp, USA).

### Indirect immunofluorescence assay (IFA)

Marc-145 cells grown in 96-well plates were infected with the recombinant or parental virus preparations (0.01 MOI) for 36 h. After fixation using 4 % paraformaldehyde, cells were washed with PBS and incubated with 6D10, a monoclonal antibody to PRRSV N protein (produced in our lab), or a goat anti-porcine IL-4 polyclonal Ab (R&D Systems, MN) for 1 h. Cells were then washed with PBS and incubated for 1 h with a Cy3-conjugated goat anti-mouse secondary antibody (Jackson ImmunoResearch Inc, USA) or an Alexa Fluor 488-labeled donkey anti-goat secondary antibody (Abcam, UK), respectively. Images were taken using a Leica confocal microscope.

### Western Blotting

Marc-145 cells were infected with the recombinant or parental viruses (0.01 MOI) for 36 h. The cells were then lysed in RIPA lysis buffer (Beyotime, China). Cell lysates were electrophoresed on 12 % SDS-PAGE gels and transferred onto PVDF membranes (Millipore, USA). The membranes were blocked and incubated for 1 h with 6D10 or a goat anti-porcine IL-4 polyclonal Ab (R&D Systems, MN), then incubated with HRP-conjugated goat anti-mouse IgG (Sigma-Aldrich, USA), or HRP-labeled donkey anti-goat IgG (Sigma-Aldrich), as secondary antibodies, respectively. Immunostained proteins were visualized using ECL Western Blotting Substrate (Beijing Cowin Bioscience, China).

### Genetic stability of the recombinant PRRSV

To investigate whether the inserted pIL-4 gene could be stably maintained in the recombinant virus, the virus was serially passaged 15 times in Marc-145 cells. Viral RNA was isolated from cells infected with different passaged recombinant viruses (P5, P10 and P15) and RT-PCR was performed using specific primers (Table 1). PCR products were cloned into the pEASY™-Blunt Simple Cloning Vector (TransGen Biotech, China) and the DNA was sequenced.

### Animals, vaccination, and HP-PRRSV challenge

Twenty four-week-old piglets were obtained from a PRRS-free farm in Harbin, China. All piglets were negative for anti-PRRSV antibodies, as assessed by commercial ELISA kit (IDEXX, USA) and PRRSV-free in serum by RT-PCR. Animals were randomly allotted to four groups and housed separately. Piglets were intramuscularly immunized twice with 2 mL (1 × 10^5.5^ TCID_50_/mL) of recombinant virus (group 1) or parental virus (group 2) on 0 and 14 DPI.

All piglets were challenged intranasally with 2 mL HP-PRRSV (HuN4-F5, 1 × 10^5.0^ TCID_50_/mL) at 28 DPI, except those in Group 4, which were given only cell culture medium (uninfected control). Rectal temperatures were documented daily after challenge. Pigs were monitored daily for clinical signs, including dyspnea, lethargy, cough and anorexia. The pigs were euthanized at 28 days post-challenge (DPC). The severity of the clinical signs was evaluated daily after challenge as reported [53]. Briefly, scores ranged from 1 to 4 were determined for behavior, respiration and coughing. Score 1, 2 and 3 represents normal, mild and severe, respectively. Score 4 represents death. To estimate the severity of pathological lesions, gross lesions of each lobe were scored and evaluated as percentage of lung with grossly visible pneumonia [54]. Animal research was conducted under the Animal Welfare and Ethical Guidelines of the Institutional Animal Care Committee.

### Circulation antibody titration

Sera were collected at 14 and 21 DPI and at 0, 7, 14, 21, and 28 DPC. PRRSV N-specific antibodies were tested using a commercial PRRS ELISA kit (IDEXX, USA) following the manufacturer’s instructions.

### T lymphocyte proliferation assay

Peripheral blood mononuclear cells (PBMCs) were isolated from experimental pigs as previously described [55]. Briefly, The PBMCs were diluted to 5 × 10^6^/mL in RPMI 1640 supplemented with 10 % FBS and plated in 96-well flat-bottom plates at 100 μL per well in triplicate. Subsequently, the purified-PRRS virions (20 μg/mL) as stimulator were added to the cultures, and PHA (5 μg/mL, Sigma-Aldrich, USA) was used as a positive control. After incubation for 72 h at 37 °C in 5 % CO_2_, 10 μL of WST-8 (Cell Counting Kit-8, Beyotime Biotechnology, China) was added to each well and the cells were incubated for 2 h. At the end of the incubation, the absorbance was determined at 450 nm. The stimulation index (SI) was calculated as the ratio of the average optical density (OD) value of wells containing antigen-stimulated cells to the average OD value of wells containing cells cultured with medium alone.

### Flow cytometric (FCM) analysis

Flow cytometric (FCM) analysis was performed to investigate various lymphocyte populations in the blood. Briefly, 50 μL of anti-coagulated blood sample was incubated with SPRD-labeled mouse anti-pig CD3ε, FITC-labeled mouse anti-pig CD4, and PE-labeled mouse anti-pig CD8 (Southern Biotech, USA), in the dark for 30 min. 1x RBC lysis buffer (BioLegend, San Diego, CA, USA) was added to each sample and gently vortexed immediately for 15 min. Samples were centrifuged at 350 × g for 5 min and washed twice with 2 % FBS, before FCM studies were performed using a BD Accuri™ C6 flow cytometer (BD Biosciences, San Jose, CA, USA). Frequencies of lymphocyte populations were determined from 100,000 events using BD Accuri™ C6 software.

### Cytokine assays

Lymphocytes were prepared and stimulated as described above. After incubation for 72 h, culture supernatants from PBMCs of piglets were collected and Th1-type cytokine (IFN-γ) and Th2-type cytokine (IL-4) levels were assessed using commercial ELISA kits (Cloud-Clone Corp, USA).

For the detection of IFN-γ and IL-4 production in pigs at various time-points, sera samples were isolated, and cytokines concentrations were determined according to manufacturer’s instructions.

### Viremia testing

Total RNA was extracted from sera using Trizol Total RNA Extraction Kit (Shanghai Sangon Biological Engineering Technology & Services, China), cDNA was synthesized using AMV Reverse Transcriptase XL (TaKaRa, Japan) with oligo (dT) and Quantitative RT-PCR was performed as described previously [50].

### Histopathological examination

All pigs were euthanized at 28 DPC. Lungs were harvested and fixed in 10 % neutral buffered formalin then stained with hematoxylin and eosin (H&E) for histopathological examination.

### Statistical analysis

GraphPad Prism V.5 software (GraphPad Software, La Jolla, CA, USA) was used for statistical analysis. Results were considered to be statistically significant when the p value was less than 0.05, using one-way analysis of variance (ANOVA).

## Abbreviations

PRRSV: Porcine reproductive and respiratory syndrome virus
pIL-4: Porcine Interleukin-4
IFA: Indirect immunofluorescent assay
HP-PRRSV: Highly pathogenic PRRSV
DPI: Days post-immunization
DPC: Days post-challenge
FCM: Flow cytometric
TRS6: Transcription regulating sequence for ORF6
Lg: Log10
